# Hierarchical self-assembly into chiral nanostructures

**DOI:** 10.1039/d1sc03561d

**Published:** 2021-11-09

**Authors:** Yutao Sang, Minghua Liu

**Affiliations:** Beijing National Laboratory for Molecular Science (BNLMS), CAS Key Laboratory of Colloid, Interface and Chemical Thermodynamics, Institute of Chemistry, Chinese Academy of Sciences Beijing 100190 China liumh@iccas.ac.cn

## Abstract

One basic principle regulating self-assembly is associated with the asymmetry of constituent building blocks or packing models. Using asymmetry to manipulate molecular-level devices and hierarchical functional materials is a promising topic in materials sciences and supramolecular chemistry. Here, exemplified by recent major achievements in chiral hierarchical self-assembly, we show how chirality may be utilized in the design, construction and evolution of highly ordered and complex chiral nanostructures. We focus on how unique functions can be developed by the exploitation of chiral nanostructures instead of single basic units. Our perspective on the future prospects of chiral nanostructures *via* the hierarchical self-assembly strategy is also discussed.

## Abstract 2

This review shows how chirality may be used for the design, construction and evolution of higher ordered and complex chiral nanostructures through hierarchical self-assembly.

## Graphical abstract

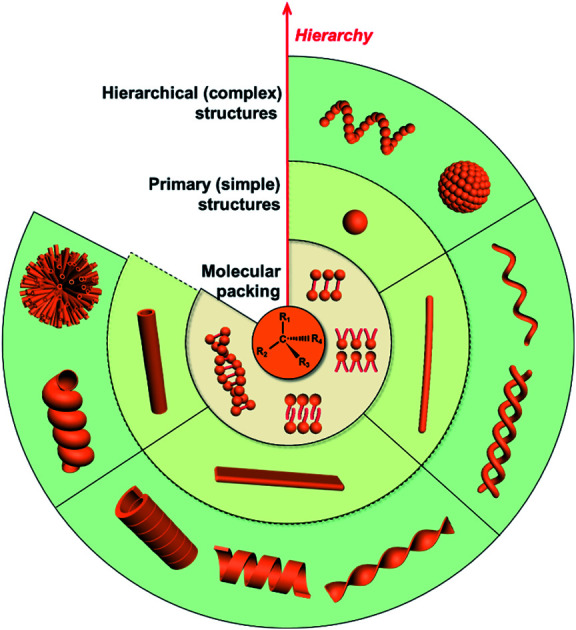

## 1. Introduction

Chirality as a fundamental property of nature is vital in many fields including chemistry, biology, physics and materials science.^1–5^ Apart from the molecular level, chirality usually occurs in more esoteric forms, such as the secondary structures of proteins, double-stranded DNA and nanoscale helices in biosystems. In addition, complex biological processes depend critically on highly ordered chiral architectures that are formed through the hierarchical self-assembly of functional building blocks in constant dynamic interactions. These natural chiral nanostructures emerge with unique and peculiar properties, and more importantly, can evolve and adapt to an external environment.

**Fig. 1:**
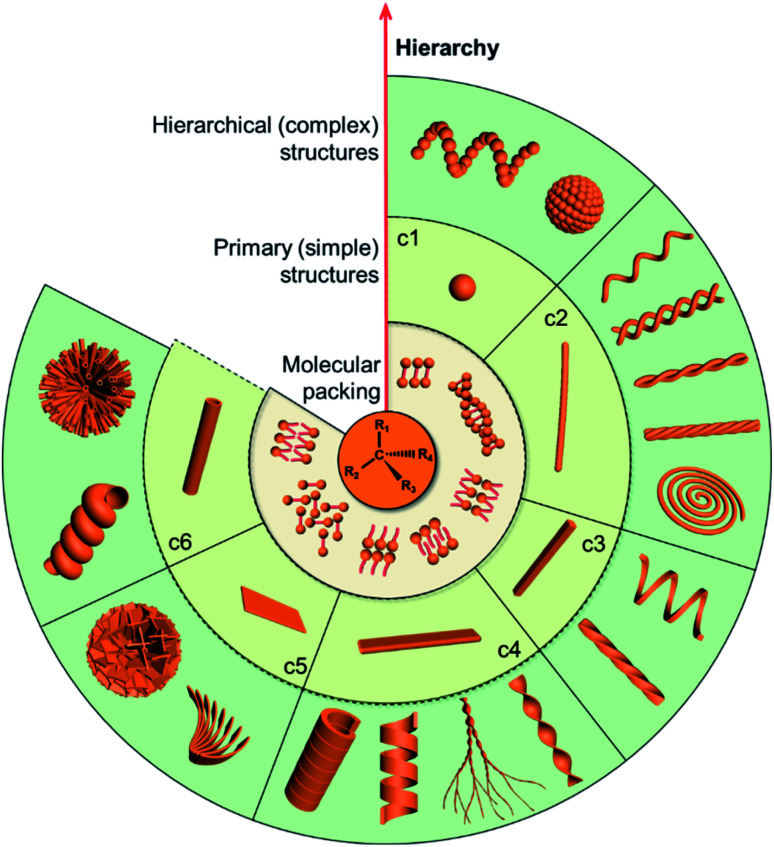
The diversity of chiral nanostructures constructed through hierarchical self-assembly. Starting from the molecular level, various primary structures can be formed through plausible packing modes. Hierarchical self-assembly of higher order or complex structures can be attained by using these primary nanostructures as building blocks. In the clockwise direction, (c1) zero-dimensional nanospheres can form helical sphere chains and microspheres; (c2) one-dimensional nanofibers can further form single-helical fibres, double-helical fibres, helical bundles with two fibres and multiple fibres, and chiral spirals; (c3) one-dimensional nanorods can assemble into helical and twisted nanorods; (c4) two-dimensional nanobelts can form twisted ribbons, dendritic twists, helical ribbons, and chiral tubules; (c5) two-dimensional nanosheets can assemble into helically arranged petals and microspheres; (c6) three-dimensional nanotubes can form microtubule flowers and helical tubes.

Motivated by the hierarchical nanostructures exhibited by nature, much effort has been devoted to the fabrication of supramolecular chiral materials with superior properties, such as asymmetric synthesis in pharmaceutics,^6–8^ chiral recognition,^9,10^ separation,^11,12^ switches,^13^ optics and electronics.^14,15^ As depicted in Fig. 1, diverse chiral nanostructures can be formed by hierarchical self-assembly strategies. The design and properties of building blocks are the core for hierarchical self-assembly into chiral nanostructures. Generally, the introduced chiral element of molecules or units determines the final supramolecular chirality, while the balance between different interactions directs the hierarchical self-assembly. Both of them are crucial for the chiral expression on different scales and corresponding functions. In terms of geometry, the chiral nanostructures formed at the initial stage can be one-dimensional (1D) fibres and nanorods, two-dimensional (2D) nanobelts, nanosheets, and three-dimensional (3D) nanotubes, which extremely depends on the interactions between the units. These primary nanostructures may serve as building blocks and further self-assemble into complex assemblies of higher orders. Therefore, hierarchical nanostructures could be considered as the evolution of primary nanostructures and not as complicated as they seem to be, since most of them indeed come from the curving or twisting of basic nanostructures. For example, 1D nanofibers can curve or align along a certain direction to form nanospirals; they can also bind together and form single helical, double helical or super-helical nanostructures (Fig. 1). Another example is nanobelts, which can present saddle-like curvature (namely chiral twists) or cylindrical curvature (helical ribbons) in their structures.^16^ Generally, chiral twists are thermodynamically stable, whereas helices are unstable and may eventually evolve into tubules. Not only the interactions between building blocks but also the external environment can affect the evolution of planar structures into chiral twists or helical structures.

Chirality is one of the basic mechanisms that guides the assembly of constituent building blocks into nanostructures.^17–20^ Using asymmetry to manipulate molecular-level devices and macroscopically functional materials is a major challenge in materials science and supramolecular chemistry. During such a process, the hierarchical chirality transfer among multiscale levels plays a vital role.^5,21^ However, how the molecular asymmetry is translated between different scales remains largely not well understood, and a fundamental review of the underlying processes is required. Herein, concise foundational rules and strategies for rational design and fabrication are provided in this review. Recent major achievements in the development of hierarchical self-assembly are also highlighted. In particular, the manipulation of chiral hierarchical self-assembly and the corresponding energy change is discussed with specific examples. The novel applications of chiral nanostructures in optics and electronics as well as bio-related functions are also summarized. Finally, we discuss the challenges and promising research focuses for the development of next-generation chiral nanostructures *via* hierarchical self-assembly.

## 2. Hierarchical chiral structures in nature

**Fig. 2:**
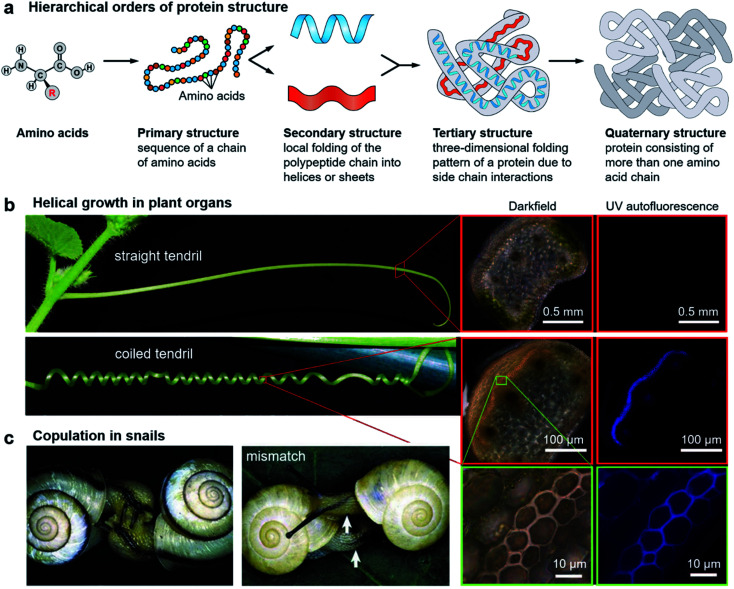
Hierarchical chiral structures in nature. (a) Hierarchical order of protein structures. Starting from amino acids, protein structures can be classified in terms of four different levels: primary, secondary, tertiary, and quaternary. (b) Images of a straight tendril and coiled tendrils, and darkfield and UV autofluorescence of the tendril cross. Darkfield and UV autofluorescence of a straight tendril show no lignin signal, while the coiled tendrils show strong lignification in the fibre ribbon. The increased magnification reveals that ventral cells are more lignified than dorsal cells. Reproduced from ref. 29 with permission from American Association for the Advancement of Science, copyright 2012. (c) Successful two-way copulation between the dexters of *E. congenital* and genital mismatch between a sinistral variant (right) and an ordinary dexter (left) of *Bradybaena similaris*. Arrows indicate genital openings that cannot be joined. Reproduced from ref. 44 with permission from Springer Nature, copyright 2003.

It is abundantly clear that chirality has an irreplaceable effect in the biological context, and nature has evolved with a common chiral sense termed biological homochirality. Therefore, chirality is often used to guide biological processes and tune living functions. Specifically, many hierarchical structures in nature exhibit chiral features. Moreover, the origin of chirality is closely related to the life's start. Hence, in order to reveal the mystery of chirality and draw inspiration from nature, a better understanding of chirality transfer and expression on different scales is required. In this section, we discuss three typical examples of chiral structures at different levels in nature: the fabrication of protein structures, helical growth in plants and copulation in snails (Fig. 2).

### 2.1 Hierarchical orders of protein structures

As an elegant and complex structure in nature, proteins are polypeptide chains consisting of different amino acid residues bonded in a definite conformation. The formation of a protein structure is a classic example of a hierarchical self-assembly process. It is well admitted that the structure of proteins is categorized in terms of four levels: primary, secondary, tertiary, and quaternary (Fig. 2a).^22^

The primary structure of proteins is the sequence of a chain of amino acids. Except glycine, all of the amino acids are chiral. Moreover, natural proteins contain only l-amino acids. Such handedness has far-reaching impacts on the hierarchical structures of proteins. On the other hand, the sequence of amino acids is unique for a particular protein and plays important roles in specific 3D structures and subsequent biological functions. The secondary structure is the local folding of the above polypeptide chain into two common conformations: the right-handed α-helix and β-pleated sheet. The coiled structure of α-helices is mainly stabilized by intrachain hydrogen bonds, while the β-sheets are mostly stabilized by hydrogen bonding between polypeptide strands.

The next level of the protein structure is the tertiary structure, which is a large-scale 3D folding pattern as a result of side chain interactions. Different from the secondary structure which involves only hydrogen bonding, basically all kinds of non-covalent bonds including hydrogen bonds, dipole–dipole interactions, and electrostatic and hydrophobic interactions exist in tertiary structures. In addition, disulfide bonds which belong to covalent interactions also contribute to tertiary structures. Disulfide bridges are bonds between the sulphur-containing functional groups of amino acid chains. Since the disulfide bonds are much stronger compared with other noncovalent interactions, they ensure that the polypeptides can rigidly connect to one another.

The first three levels are mainly concerned with a single polypeptide chain or subunits. The arrangement of more than one subunit gives rise to the quaternary structure of proteins.^23^ Note that not all of the proteins have the quaternary structure, and many of them contain only three levels of structures. One typical example with the quaternary structure is the blood protein haemoglobin, which has two α and two β subunits.^24^ Every subunit has a heme group, using the inside iron atoms to bind molecular oxygen. It is appreciated that the binding and release of oxygen in the body closely depend on subtle conformational transformations. For example, once one oxygen molecule is bound to one heme, it will increase the binding affinity of the remaining three hemes. This means that the binding of oxygen to the four hemes follows a cooperative process instead of binding independently. This cooperative way largely enhances the efficiency of oxygen binding, which is critical for the development of life in organisms of higher levels.^24^

It should be noted that only a folded protein (native structure) has full functions during biological processes, and it usually loses the functions once the 3D shape is destroyed.^25^ The latter case is called protein denaturation, which means that the protein loses its higher-level structures, but not its primary sequence. Temperature, pH or exposure to chemicals can denature proteins. Denaturation can be reversed for some proteins, while it can also be permanent such as the fried eggs.

The above hierarchy of protein structures is illustrated in Fig. 2a. As we have seen, each level of the structure plays different roles, and the final function relies on its own unique shape. This example shows the importance of hierarchical nanostructures from the molecular level to the supramolecular nanoscale.

### 2.2 Helical growth in plant organs

A variety of plants in nature exhibit certain degrees of handedness. Perhaps the most commonly observed phenomenon is the growth of climbing plants by coiling around a support.^26–30^ For example, Fig. 2b shows the helical growth morphology of tendrils.^29^ The major function of tendrils is to provide an independently stable structural support, thus enabling the plant to get more sunlight and other favourable sources. During growth, the tendril is initially straight before it finds and touches a support. In many species, there is a touch-sensitive region on the tip of tendrils. Once this region encounters a suitable support, twisting around the object will start within seconds or minutes. In other cases, the full circumference is sensitive and coiling starts from the initial contact direction. Different from the stems of twining plants, the coiling direction is controlled by the orientation of the initial contact instead of pre-existing helical sense in the tendril itself, that is, the tendril coils around the support can be either left-handed or right-handed.

Furthermore, many climbing plants also form a spring-like structure between the anchored tip and the plant itself.^31^ Note that the handedness near the anchored tip and plant are opposite, and the reversed point, which now called ‘perversion’, is usually located in the centre of the tendril (Fig. 2b). As a result, the tendril coils reduce the distance from the support and also enhance the flexibility when encountering wind or other stresses.^29^

The basic mechanism of tendril coiling was recently explored,^32–34^ and the structural features during coiling were observed by microscopy (Fig. 2b).^29^ Compared to straight tendrils, stiff and lignified gelatinous fibre (g-fibre) cells were clearly visible both in darkfield microscopy and UV autofluorescence. Such a kind of g-fibre cell has been found also in reaction wood, which serves as a structural support and forces to straighten and lift growing branches. As depicted in magnified windows, the fibre ribbon has a bilayer nanostructure, with the inner wall layer showing more lignification relative to the outer layer. Due to the differential contraction of this bilayer structure during coiling, the inner lignified cells can shrink and compress more than the outer layer, resulting in the occurrence of helical growth. Similarly, this interaction causes the formation of coils between the plants and attachment point, while the perversion and the opposite handedness can be explained by the overwinding of the coils when both ends are pulled further apart.^29^

It should be noted that there are some cases in which the handedness of the helical pattern is fixed in the same direction for all individuals, which means that their chirality is controlled. For example, the twists of *Typha domingensis* leaves and *Narcissus tazetta* are always left-handed, while *Pancratium maritimum* generally shows right-handed twists.^30,35^

### 2.3 Copulation in snails

Apart from plants, chiral structures also widely exist in living animals. Many birds, sea organisms, and butterflies exhibit unique colours due to the interaction between light and their nanostructures, which are imprinted on their exoskeletons.^36–38^ Some certain species of beetles are known to have the ability to reflect circularly polarized light. For example, Beetle plusiotis gloriosa (or Chrysina gloriosa) can reflect only left-handed circularly polarized light, showing a brilliant metallic appearance.^39,40^ However, such a reflection disappears when observed with a right circular polarizer. The selective reflection of a single-handed circularly polarized light is due to the helical microstructure on the beetle surface, which functions in a similar way to the optical response of cholesteric liquid crystals (focal conic domains).^41^ Indeed, optical devices and photonics that benefit from the mechanism of beetles have been developed.^42,43^

Another vivid example of morphological chirality at the macroscale is snails. The direction of spiralling shells seems to play a vital role in the species propagation. As described in Fig. 2c, copulation becomes much more difficult when the two shells have opposite chiral rotations.^44^ Therefore, the chiral minority of shells is eliminated due to the homochirality controlled species propagation. In fact, the chiral status of the shells is complicated between different species, and even within one species in different places.^45–47^ The formation and mineralization of mollusk shells has been developing rapidly because of the well-designed morphologies and hierarchical structures of biominerals, which provide versatile properties. The emerged architectures from self-assembly under ambient conditions provide a sophisticated model for materials science, such as nacre.^48^ In snail shell formation, crystallite column fibers firstly form lath, folia, or crystalline lamellae, and then these structures further assemble into various microstructures including crossed foliated lamellar, lamello-fibrillar, foliated aragonite, fibrous foliated, and isolated tablet structures.^49–52^ Although enough information has not been accumulated so far to understand the chirality origin of snails, the latest investigation suggested that soluble mollusc-shell proteins and organic membranes determined the pattern of hierarchical biomineral growth.^53–55^

## 3. Rational design and fabrication strategies

**Fig. 3:**
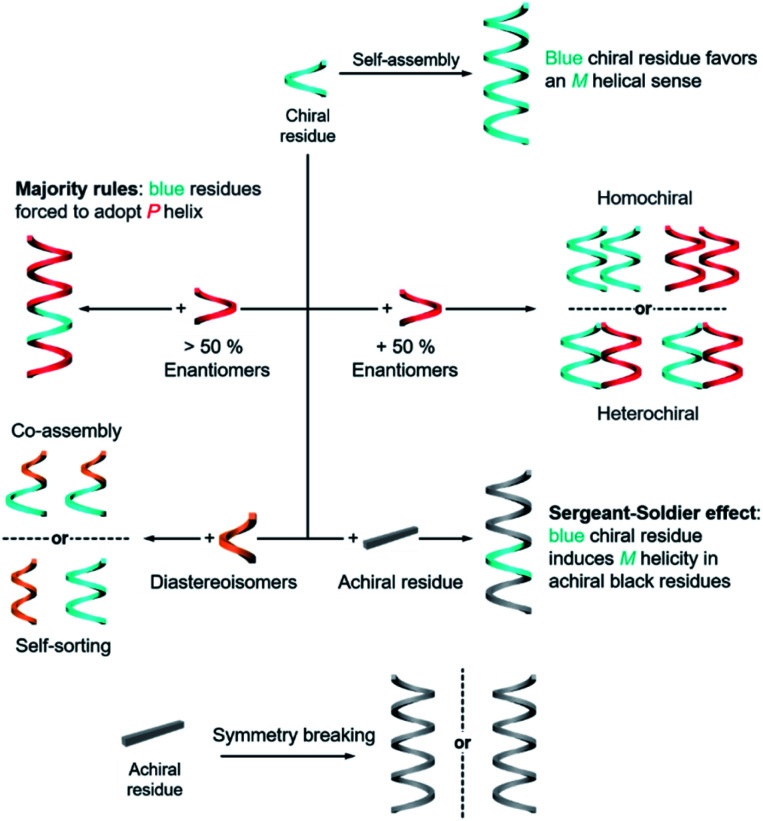
Fabrication and transmission of chirality during hierarchical self-assembly.

The rational design of building blocks is fundamentally important for hierarchical self-assembly. Although the transformation and switching of as-formed nanostructures can occur, the post-modification or morphology change also largely depends on the properties of basic units. In this section, we discuss the rational introduction of chirality and mechanisms/rules of chirality transmission, such as control of chirality *via* hierarchical levels and the amplification of chiroptical signals (Fig. 3).

The design of molecules or building blocks is a subtle balance. Several factors, such as the position of chiral centre/axis and the type of noncovalent or covalent interactions, should be considered. Notice that the assemblies usually have more than one interaction. Particularly, the introduction of asymmetry sense can dictate the overall conformation of nanostructures and also the related supramolecular chirality. Generally, chiral nanostructures can be prepared from enantiopure chiral residues, and the chirality is transited from monomers to aggregates of higher levels. This is probably the simplest way of chiral transfer, while two or more component-based systems are more sophisticated (Fig. 3). If the mixing ratio of two enantiomers is 1, which means that the enantiomeric excess of the whole system is zero, two kinds of aggregates may form with a difference in energy.^56^ When the interaction of the same chiral residues is stronger, two homochiral domains (sometimes called conglomerate) are preferentially formed. Such a kind of system is actually a mechanical mixture of enantiomerically pure aggregates. In contrast, if the opposite enantiomers have a greater affinity than the same enantiomer, heterochiral domains (also called the racemic compound or true racemate) will form. Generally, the homochiral case is a lower-energy state, while the final energy state of heterochiral is higher. The above-mentioned chiral discrimination is easier to realize in the solid state like crystals.

When the mixing ratio of two enantiomers is not equal, which means that the enantiomeric excess is not zero, the final helical sense of the whole aggregate prefers the chirality of the enantiomer of a higher amount. This discipline is called the ‘majority rules’ effect (Fig. 3). Apart from chiral residues, achiral components can also form chiral nanostructures when mixed with chiral components, following the so-called ‘sergeants and soldiers’ effect. In such a case, the achiral units, considered as ‘soldiers’, obey the chirality of a small number of chiral molecules (the ‘sergeants’). It is noticed that both majority rules and sergeants and soldiers effects describe the amplification of a small chiral bias at the monomer level to a higher-order level, and this amplification of chirality is a nonlinear response. Both of these effects are originally proposed by Green and co-workers in polymers with covalent bonds^57,58^ and are now widely observed in non-covalent systems, on 2D surfaces and larger 3D structures as well.^5,20,59–65^

The hierarchical self-assembly of structurally similar analogues is another important approach to achieving chiral nanostructures. The self-assembly of analogous mixtures generally results in two distinguished aggregation states, namely, self-sorting and co-assembly (Fig. 3).^66,67^ Co-assembly requires similar packing units or relatively strong interaction between analogous units. Therefore, different basic units can combine and form chiral nanostructures together. Meanwhile self-sorting represents the process of monomers to recognise self- and/or non-self-species within a complex mixture and further selectively self-assemble with their homogenous units.^68^ Typically, the self-sorting process depends on the same intermolecular forces that direct the discrimination and recognition process. It should be noted that the self-sorting behaviour is ubiquitous in nature and is closely related to many biological processes such as DNA replication and transcription.

In addition to the above discussion with chiral molecules or monomers that are inherently chiral, completely achiral building blocks can also form chiral nanostructures when the packing model is asymmetrical (Fig. 3).^69^ Such a phenomenon is called symmetry breaking, which has been observed in many materials including crystals,^70,71^ liquid/solid interface,^64,72^ liquid crystals,^73–75^ Langmuir monolayers,^76–78^ supramolecular gels,^79–81^*etc.* Normally, the chiral sign of aggregates from exclusive achiral components is randomly distributed, but the emerging chirality during symmetry breaking can be control by either chemical ways such as sergeants and soldiers effect or physical fields such as circularly polarized light,^82,83^ hydrodynamic vortex,^79,84,85^ and rotational and magnetic force.^86^ Symmetry breaking of achiral units not only provides an intriguing way to prepare nanostructures with macroscopic chirality or optical activity instead of synthesis and purification of chiral enantiomers, but also contributes to the understanding of the emergence of initial chiral bias and homochirality in nature.

The above-mentioned principles are general and also apply to inorganic building blocks such as carbon nanotubes, graphene oxide, nanoparticles and any interactions between chiral species.

## 4. Manipulation of hierarchical chiral self-assembly

**Fig. 4:**
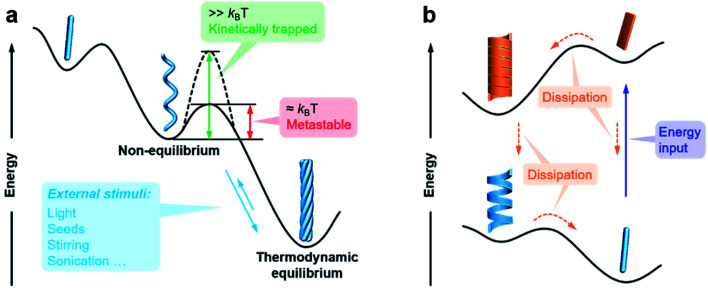
Energy landscapes illustrating the formation and transformation of different chiral nanostructures through hierarchical self-assembly. (a) Non-equilibrium, thermodynamic equilibrium, and (b) far-from-equilibrium self-assembly.

The previous section briefly discussed the transmission and amplification of chirality between multiple scales. In fact, all of these effects and mechanisms can be understood from the energetic point of view. It should be noted that there is a very small energetic difference between two enantiomers due to charge parity violation, called parity violating energy difference (PVED). The best predictions for the expected PVED values is in the range of 10^−18^ to 10^−14^ kJ mol^−1^.^87^ However, this value is too small to exert any influence either on the change of the equilibrium constant or on the asymmetric autocatalytic chemical reaction.^88^ Therefore, we generally consider that there is not energetic difference between two enantiomers when they independently interact or assemble with achiral molecules. When two enantiomers interact with another chiral component, the energy becomes different for these two interactions, and thus their assembling rate and chiral nanostructures are different, resulting in different properties and functions. This principle is general for many cases and also applies to the manipulation of hierarchical self-assembly into different chiral nanostructures. Fig. 4 shows the energy landscape of the formation and transformation of different chiral nanostructures.

The formation of complex chiral nanostructures usually goes through several stages (see Fig. 1). Due to the relatively weak non-covalent interactions and the dynamic features, traditional supramolecular chemistry including the study of chiral nanostructures mainly focuses on systems at equilibrium.^89^ During the thermodynamically equilibrated self-assembly, metastable chiral assemblies may be formed firstly, but finally transform into a more thermodynamically stable state with complex chiral structures. Thermodynamically equilibrated nanostructures can persist for a long time due to their thermodynamic stability. In this case, the evolution of chiral nanostructures occurs without any external stimulations because the energy barrier of the metastable state is around *k*_B_T, and thus it can be overcome at room temperature on an experimentally observable time scale usually from minutes to several months.

In contrast, if the energy barrier of a non-equilibrium state is much higher, the chiral structures are temporarily stable, and the whole system is trapped in a local energy minimum (Fig. 4).^90–95^ It would take a very long time to convert to a more stable state. External physical stimuli, such as sonication, stirring and addition of seeds, can accelerate the interconversion by eliminating the energy barriers.^96–98^

The so-called far-from-equilibrium contrasts starkly with the mode of thermodynamically driven assembly processes (Fig. 4b). Different from downhill self-assembly, far-from-equilibrium systems require a continuous supply of energy to maintain their structures.^99,100^ Once the energy supply stops, such a kind of system would fall apart and convert to a thermodynamically minimum state (or a kinetically trapped state, if present).^101^ In fact, many advanced processes and functions, such as biological machinery and cell biology, require continuous free energy to maintain assemblies in the functional state and activate chemical reactions so that they can sustainably work.^102^ Life can be regarded as a far-from-equilibrium system. Moreover, continuous energy-driven transformation makes it possible to construct more elaborate structures with devious functions.^103^

It is worth noting that a system in thermodynamic equilibrium conditions may still be dynamic. Although the overall system does not change over time, monomers continuously assemble and disassemble at a certain rate. Therefore, if the nanostructures or building blocks contain sensitive functional groups, external environment changes, such as pH, polarity of solvent, light irradiation (for example cyclization and *cis*–*trans* isomerism), or adding another species (no matter chiral or achiral), can bring the system to new thermodynamic equilibrium conditions.^13,98,104,105^

The above discussion concludes that the pathway complexity of self-assembly enables diverse and hierarchical chiral nanostructures, while the dynamic nature of supramolecular chemistry allows the transformation of chiral nanostructures among different states.^13,106^ In the following of this section, hierarchical self-assembly and transformation of chiral nanostructures are illustrated by typical recent examples. The chiral nanostructures of emerging materials, such as metal–organic frameworks (MOFs), covalent organic frameworks (COFs) and chiral perovskites, are also discussed.

### 4.1 Pathway complexity in hierarchical self-assembly

It is well admitted that 1D supramolecular polymerization follows either cooperative (also known as nucleation–elongation) or isodesmic mechanisms.^107,108^ These mechanisms of noncovalent ensembles are respectively analogous to the chain and step-growth mechanisms of covalent polymers.^108^ In terms of energy, cooperative assembly can be divided into a less favourable nucleation process and subsequent highly favourable elongation process.^109,110^ Apparently, equilibrium constants for these two stages are different. In contrast, all binding constants throughout the isodesmic mechanism are equal. Recent achievements indicate that the kinetic-pathway-controlled supramolecular polymerization gives rise to highly ordered chiral nanostructures with sometimes unpredictable and emergent functions.^94,99,101,102^

**Fig. 5:**
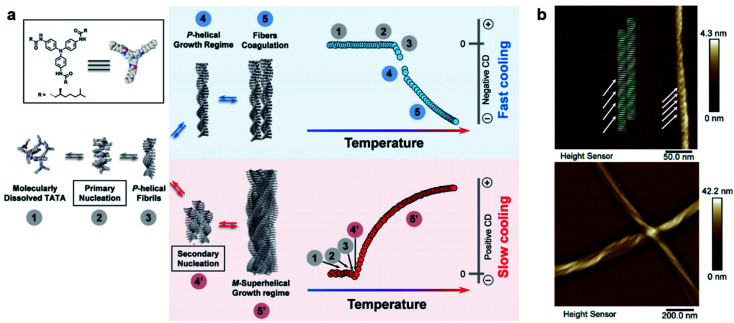
(a) Schematic representation of the two self-assembly pathways depending on the cooling rate. Note that assemblies have the same chirality in step 3, indicating that the primary aggregates have identical columnar packing for both cooling regimes. (b) Atomic force microscopy (AFM) images of self-assemblies through fast cooling (10 K min^−1^, top panel) and slow cooling (1 K min^−1^, bottom panel). Reproduced from ref. 111 with permission from Wiley-VCH, copyright 2019.

Fig. 5 shows an intriguing case that hierarchical supramolecular structures and chirality can be simply controlled by the cooling rate of initial solutions.^111^ When the hot solution of molecules was cooled from 50 to 20 °C at a cooling rate of 10 K min^−1^ (referred as fast cooling), the assemblies showed some coagulations of single *P*-helices with a negative circular dichroism (CD) signal. Meanwhile for slow cooling (1 K min^−1^), a global *M*-superhelix internally composed of individual *P*-helices was observed (Fig. 5b), and the corresponding CD signal became positive. The concentration effect on kinetic aggregation was also investigated. At low concentrations, single fibrils of *P* helicity and their coagulation dominated the self-assembly process. At high concentrations, the short *P* helices further self-assembled into superhelices with *M* helicity. The inversion of the CD signal at high concentrations was due to the highly twisted bundles of supramolecular single fibres. At medium concentrations, the self-assembly pathway depends on the cooling rate. In other words, the short *P*-helical fibrils generated from primary nucleation were shared in both pathways, but these fibrils evolved through primary growth to form longer fibrils and coagulation with the same helicity (for fast cooling), or through secondary nucleation and growth to form *M*-superhelical fibres (for slow cooling).^111^

### 4.2 External change controlled hierarchical self-assembly

The precise control of chiral nanostructures is the core challenge at the heart of hierarchical self-assembly. Compared to external physical stimuli (such as sonication, stirring, *etc.*), environmental changes (chemical stimuli) provide more possibilities to manipulate chiral nanostructures with precise helical pitches, diameters, and handedness.

**Fig. 6:**
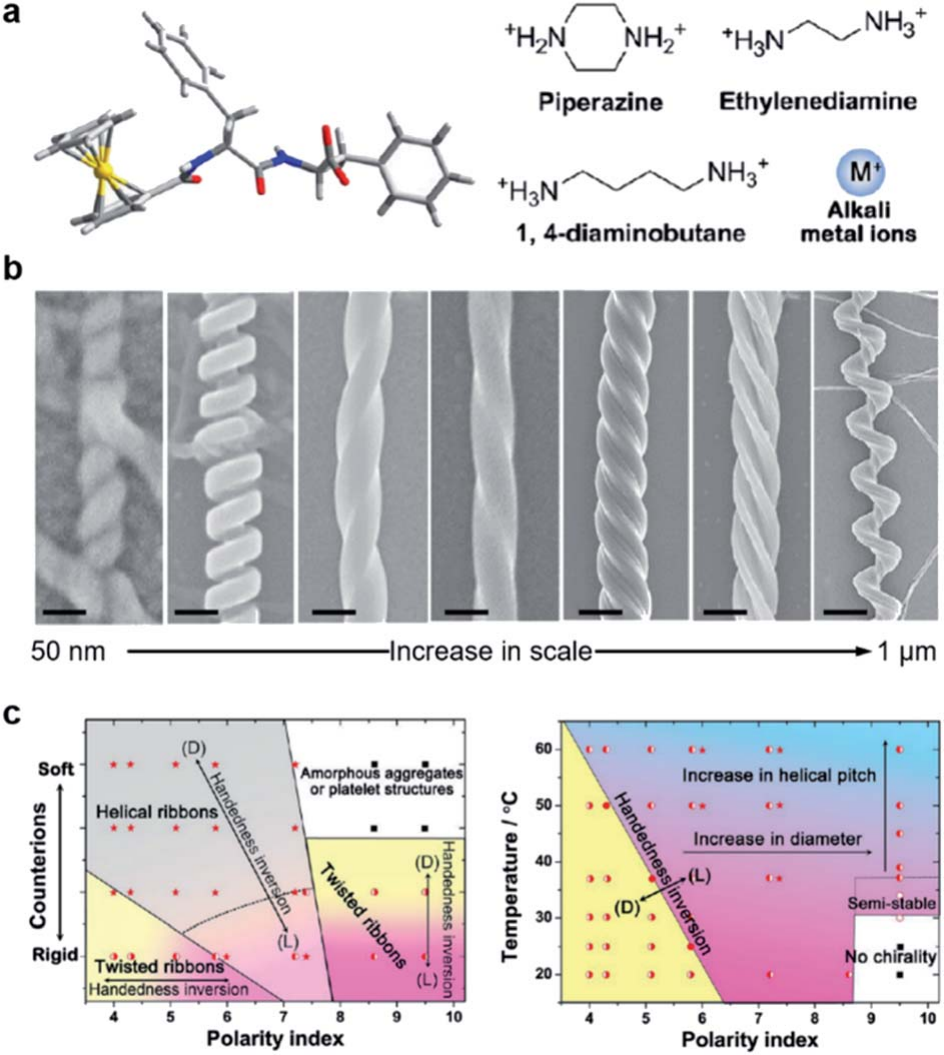
Hierarchical and chiral nanostructures with varied forms using a combination of basic strategies. (a) Molecular structure of ferrocene (Fc)-modified dipeptide and counter ions used during chiral self-assembly. (b) Rationally designed hierarchical chiral nanostructures (left-right): tube-like helical ribbons (scale bar = 50 nm), rigid nanosprings (scale bar = 100 nm), right-handed twisted ribbons (scale bar = 100 nm), left-handed twisted ribbons (scale bar = 100 nm), big twists (scale bar = 200 nm), nanohelices formed *via* the twist of two crossed ribbons (scale bar = 300 nm) and nanoscrews (scale bar = 1 μm). From left to right, the tube-like helical ribbons were self-assembled in the presence of alkali metal ions (Na^+^ or K^+^) in less polar solvents; the rigid nanosprings were grown with ethylenediamine or 1,4-diaminobutane in less polar solvents; the last five chiral nanostructures were self-assembled with piperazine counter ions *via* control of temperatures, solvents and pH. (c) Phase diagram for the chiral self-assembly of Fc-FF based on changes in counter ions and solvent polarity. (b) Practical “handbook” for the rational control of the architecture of the self-assembled chiral nanostructures. Reproduced from ref. 112 with permission from American Chemical Society, copyright 2015.

Fig. 6 shows a classic example of the rational design of chiral nanostructures through hierarchical self-assembly of a ferrocene (Fc)-modified dipeptide, ferrocene-l-Phe-l-Phe-OH (Fc-FF).^112^ Inspired by the regulation of β-sheet helical twists in different stages of peptide self-assembly, three basic strategies were explored to control the chiral self-assembly: counterions triggered twisting, temperature-depended kinetic effect, and solvent-regulated noncovalent interactions. As shown in Fig. 6b, various chiral nanostructures with different scales including nanoscrews, nanohelices (formed *via* the twist of two crossed ribbons), big twists (diameters of ∼250 nm and helical pitches of ∼600 nm), left- and right-handed twisted ribbons, rigid nanosprings and tube-like helical ribbons were formed by changing the counterions, solvents and temperature. The counterions and solvent polarity controlled the distinct forms of chiral structures as described in Fig. 6c. The rational control of the parameters of self-assembled chiral nanostructures, such as handedness, diameters, and helical pitches, was also summarized based on the change of temperature and solvents (Fig. 6c). Self-assembly at a lower temperature and a lower solvent polarity gave rise to stable right-handed chiral nanostructures while the increase of temperature induced handedness inversion from right to left. No chiral nanostructures were formed at a higher solvent polarity and a lower temperature, but a slight increase of temperature (>37 °C) induced the formation of left-handed chiral nanostructures. In the end, the subtle change of solvent polarity and temperature could precisely control the diameter and helical pitch of the chiral nanostructures (Fig. 6c).

### 4.3 Dissipative out-of-equilibrium hierarchical self-assembly

**Fig. 7:**
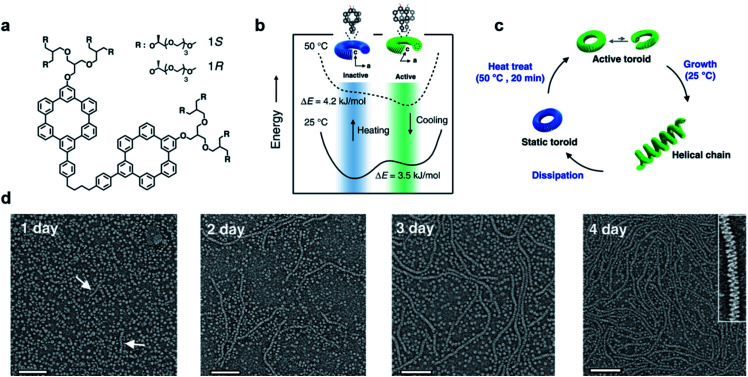
Dissipative helical polymers fuelled by heat stress. (a) Molecular structure of an aromatic macrocycle. (b) Energy diagrams of eclipsed and slipped conformations at different temperatures. (c) Schematic representation of the helical polymerization and depolymerization cycle of the toroids upon heat treatment. (d) Time-dependent negatively stained transmission electron microscopy (TEM) images after heat treatment. Scale bar, 100 nm. The arrows in the one-day image indicate the formation of short helices. Inset of the 4 days image shows a magnified image exhibiting a right-handed helical structure. Reproduced from ref. 121 with permission from Nature Publishing Group, copyright 2019.

Most of the synthetic chiral nanostructures exist in a global minimum state (thermodynamic equilibrium),^18,113,114^ while living systems in nature generally require continuous energy input to maintain their functions.^115–118^ Therefore, designing artificial assemblies with out-of-equilibrium features provides an approach to creating new properties beyond static systems.^103,119,120^Fig. 7 shows that heat stress is used as an energy source to trigger autonomous helical propagation with mechanical action.^121^

Without any energy input, uniform donut-like objects were formed in solution with high stability (remained unchanged for 2 years). Upon heating at 50 °C for 20 min, the increased cross-sectional area and hydrophobic interactions caused slipping between one another (Fig. 7b). This slipped conformation thus activated the donut-like structure, and permitted the system to evolve into chiral structures of a higher order (Fig. 7c). After the heating process and cooling to 25 °C, the assemblies maintained the toroidal structures without obvious changes. After 24 hours, the closed toroids spirally opened to nucleate helical growth and formed short helical chains. These short chains kept growing rapidly and reached the maximum lengths of nearly a micrometre in 4 days (Fig. 7d). The magnified TEM image revealed that the formed polymer chains were right-handed helical structures. Moreover, the diameter of this helix was 12 nm, which was identical to that of the individual toroids, confirming that the formation of helical chains was initiated by the open spiral. After 5 days, the length of helical polymers became short in an irregular way and collapsed to the intact toroidal structures after 12 days. Note that this whole process was totally reproducible. Moreover, if heat treatment was performed every 4 days before depolymerisation started, the helical structures and CD intensity could be maintained without decay. The mechanism of this heat-directed morphological change is illustrated in Fig. 7c. To release the system energy increased by heat stress, the toroids became activated and further helically grew to form hierarchical structures. As the thermal energy was consumed over time, the helical chain started to move towards an equilibrium state (toroid structures) by releasing energy. Finally, the energy stored in the helical structures was dissipated when the helical chains autonomously collapsed.^121^ The polymerization–depolymerization cycle of toroids also caused the reversible elongation of spherical vesicles if these toroids were encapsulated inside the lipid vesicles, suggesting the potential application in converting external stress into mechanical work.

### 4.4 Hierarchical self-assembly into chiral complex nanostructures

**Fig. 8:**
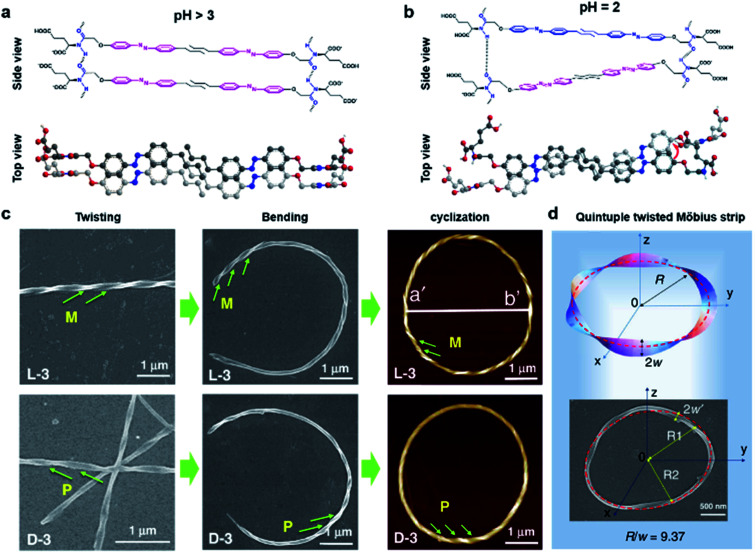
Bending and cyclization processes of twisted nanofibers. (a) When pH > 3, a bilayer structure was formed through azobenzene π–π stacking and hydrogen bonding between amide groups. Tiny molecular length difference induced straight twisted fibres. (b) When pH = 2, a counterclockwise bilayer stacking was formed due to the obvious molecular length difference. (c) Formation of chiral toroidal fibres from linear left-handed (up) and right handed (down) twisted fibres. (d) Quintuple twisted Möbius strip. *R* represents the radius of the Möbius strip, *R*_1_ and *R*_2_ represent the long radius and short radius of elliptic Möbius strip, and *w* and *w*′ represent the half-width of Möbius strip band. Reproduced from ref. 122 with permission from Nature Publishing Group, copyright 2020.

Fig. 8 shows another type of toroid nanostructure, which is composed of twisted fibres.^122^ It is worth noting that the twist number of the toroid nanostructures is important. For example, if the twist number is odd, the toroid morphology belongs to a special physical topology called Möbius strip.^123^ The Möbius strip is the simplest non-orientable surface and contains only a single edge and a single side.^124,125^ Actually, several kinds of topological structures, such as rotaxane, knot, and catenane, have been realized *via* organic synthesis.^126–128^ However, their supramolecular analogues fabricated from hierarchical self-assembly strategies are rarely reported.

Because of the existed two carboxylic acid groups in the molecular structure, the building blocks show different ionization levels at varied pH values. Density functional theory (DFT) calculation results showed that the molecular lengths were similar (2.22–2.25 nm) when pH > 3, and this tiny molecular length difference induced straight twisted fibres (Fig. 8a). However, the major components changed at pH = 2, and the molecular lengths ranged from 2.02 nm to 2.25 nm, resulting in counterclockwise bilayer stacking (Fig. 8b). As a result, the bending of twisted fibres was achieved by adjusting the pH of the system. On the other hand, the cyclization of bent fibres was much more difficult because the elongation direction of the fibres was generally random in aqueous media. One practical way to increase the probability of intra-fibre end-to-end cyclization was decreasing the concentration of building blocks in order to shorten the length and reduce the number of twisted fibres. After optimization, a concentration of 50 μM corresponded to the largest number of chiral toroidal structures (50%). Therefore, the combination of lowering pH and concentration successfully bent the straight twisted fibres, which further cyclized to form chiral toroid structures in high yields (Fig. 8c). In particular, chiral Möbius strips were obtained when the twist numbers of these nanotoroids are odd (Fig. 8d).^122^

Another noteworthy observation is the chirality control of Möbius strips. In fact, Möbius strips are chiral because of their topological structure. However, effective control of their chirality is still a challenge. In this case, the helicity of the supramolecular Möbius strips can be easily tuned by the molecular chirality of the starting building blocks.^122^

### 4.5 Helical superstructures in the bulk and thin-film states

Manipulating multiple interactions during hierarchical self-assembly is crucial for various complex biological superstructures, such as three-dimensionally folded proteins (Fig. 1a) and biologically active cell membranes. Helical superstructures have a multilevel chiral organization: the elementary units firstly assemble into ordered primary and secondary structures and then serve as building blocks for self-assembly at the next level(s). During the hierarchical assembly process, the noncovalent interactions between building blocks play a critical role in determining the morphology of the final superstructures. Despite the importance of helical superstructures in the fields of biological systems and artificial materials science, the underlying mechanisms of geometrical complexity are still puzzling.

**Fig. 9:**
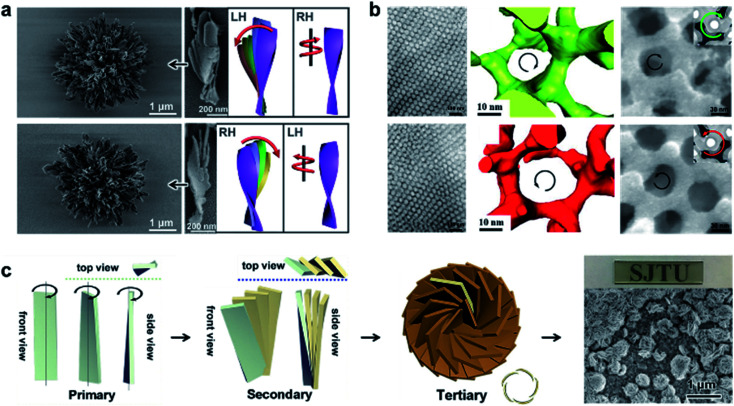
Helical superstructures in the bulk and thin-film states. (a) Gold thiolate hierarchically organized particles with l- (up) and d-cysteine (down). SEM images and corresponding schematic illustrations of the coccolith-like particles and constituent twisted ribbons. Reproduced from ref. 129 with permission from American Association for the Advancement of Science, copyright 2020. (b) TEM (left) and 3D reconstructions from electron tomography (middle) of the self-assembled polylactide-based triblock terpolymers. The right panel shows the FESEM images of the Ni network from the chirality controlled networks. Reproduced from ref. 132 with permission from American Association for the Advancement of Science, copyright 2020. (c) Schematic drawings of the hierarchical chirality and SEM images of the chiral mesostructured bismuth oxybromide (BiOBr) films. Reproduced from ref. 133 with permission from Wiley-VCH, copyright 2021.

Fig. 9a shows hierarchically organized particles with twisted spikes radially organized around a common centre.^129^ Chiral thiolates of gold in the form of nanoplatelets were firstly prepared with the help of amino acid cysteine. Such nanoplatelets act as nanoscale building blocks to form twisted nanoribbons, composed of staggered thin sheets. The assembly driving force was electrostatic repulsion, which was caused by adding organic cations, cetyltrimethylammonium bromide (CTAB), resulting in the favoured edge assembly of the nanoplatelets. Note that the nanoribbons with l-cysteine were right-handed, while the stacks of them were left-handed (see the schematic illustrations in Fig. 9a). Their handedness was proved to be controlled by the chirality of surface ligands, and mirror-imaged superstructures were observed if d-cysteine was used instead. These nanoribbons further formed spiky (coccolith-like) particles (Fig. 9a). Instead of the particles size, the evolution of multiparticle systems was found to be strongly dependent on particle symmetry and asymmetry because of the combined restrictions of anisotropic electrostatic and elastic interactions.^129^

The hierarchical self-assembly of chiral block copolymers has been proved as a facile strategy to prepare helical architectures due to the various secondary interactions of blocks.^130,131^ Through the design and synthesis of the chiral block, fascinating chiral nanostructures can be achieved with different dimensions, thus providing the possibility to study the chiral transfer on different length scales and the relationship between molecular properties and chiral self-assembly processes. For example, a recent report suggested that the bulkier chiral side groups of block copolymers were able to generate a more persistent helical bias and thus further enhance the chiral anisotropy of intersegment forces.^131^ As a result, the thermodynamic stability of the mesochiral helical morphology was increased.

If the number of homopolymer subunits increases from two to three, the synthetic triblock copolymers may form more complicated chiral nanostructures. Recently, an alternating gyroid network with controlled chirality was reported *via* the self-assembly of a chiral triblock terpolymer (Fig. 9b).^132^ Combined with small-angle X-ray scattering (SAXS) profiles, the 2D projections of TEM analysis suggested the formation of an alternating gyroid composed of a pair of gyroid networks. Due to the complicated morphology of the network phase, electron tomography and 3D reconstruction were used to visualize and characterize the chirality. Clearly, the polylactide gyroid formed from l-lactide-involving polymer showed a left-handed helical locus, while the network constructed from the polymer with opposite chirality exhibited a right-handed helical locus. The successful chirality transfer on different length scales, namely from the monomeric terpolymer to the network, was further confirmed by using the polylactide gyroid as the template for the synthesis of a chiral nickel network (Fig. 9b).^132^ Therefore, such chiral networks are promising candidates for template synthesis to prepare various chiroptical materials with well-defined nanoporosity.

One of the most compelling properties of inorganic chiral materials is optical activity. However, there are only a few cases that present colour-responsive properties towards circularly polarized light. A feasible approach to achieving this goal is the rational design of hierarchically chiral architectures with different density and dimensionality. Fig. 9c presents the fabrication process of chiral mesostructured bismuth oxybromide (BiOBr) films with three levels of chirality.^133^ The primary nanostructure is chiral nanoflakes with distorted crystal lattices at the atomic level. The chirality of the nanoflakes was mediated by chiral sugar alcohols. Subsequently, nanoplates with chiral helical stacking of nanoflakes were observed, which further acted as building blocks to form chiral vortices. As a result, the hierarchical thin film showed ultrastrong optical activities in a wide wavelength range from 350 to 2500 nm. Due to the synergistic effect of the absorption- and scattering-based optical activities, this chiral mesostructured film exhibited a significant chirality-dependent colour response towards circularly polarized light.^133^

### 4.6 Hierarchical self-assembly into macroscale chiral nanostructures

**Fig. 10:**
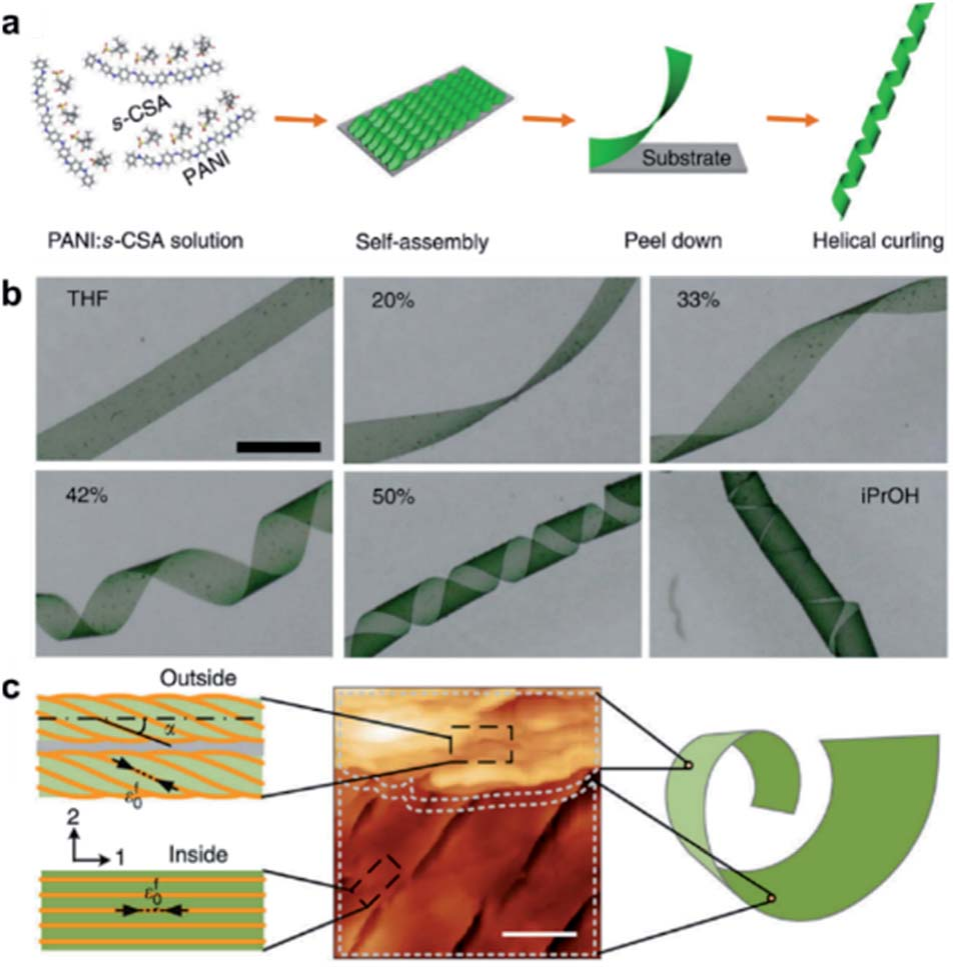
Macroscopic helical chirality generation in the stripe. (a) Illustration for the preparation of the macroscopic assemblies. (b) Photographs of the evolution of left-handed helical ribbons with increased *Φ*_iPrOH_ (the ratio of iPrOH to the total volume of iPrOH and THF). (c) AFM image of a helical ribbon; scale bar is 3 μm. The direction difference of shrinkage strain between the outside and inside surfaces inducing normal and shear strain gradients simultaneously along the thickness that in turn induces spontaneous bending curvature and torsional curvature, respectively. Reproduced from ref. 139 with permission from Nature Publishing Group, copyright 2018.

The aforementioned nanostructures are mainly at the nanoscale and microscale due to their order limitation. However, the fabrication of macroscale chiral nanostructures through hierarchical self-assembly is also possible.^134–138^Fig. 10 shows an example of how molecular chirality breaks through the scale barriers and expresses at the macroscopic level *via* a self-assembly strategy.^139^Fig. 10a illustrates the process of material preparation. Aniline monomers and chiral dopants, enantiomeric camphor sulfonic acid (CSA), were first mixed to generate single handed polymers after polymerization.

After that, these polymers further assembled on uniaxial stretched polypropylene support to form a macromembrane structure. Subsequently, with the help of tetrahydrofuran (THF), the membrane was peeled off from the substrate, thus affording flat macrostripes.

In the next step, such flat macrostripes were exposed to isopropanol (iPrOH), resulting in the formation of helical stripes. Notably, the helicity of macrostripes was well controlled by the ratio of iProH and THF proportionally (Fig. 10b). The macrostripes were almost flat in pure THF solvent, and they started to curl slightly along a single-handed helical direction when *Φ*_iPrOH_ reached 20%. The feature size of helicity further increased with higher ratios of iPrOH, and a tubular helix was eventually formed. Such helical ribbons recovered to a flat shape once re-immersed into pure THF and curled again upon re-exposure to iPrOH. On the other hand, the handedness of the helical ribbons was found to depend on the chirality of chiral dopants. Therefore, the molecular chirality of enantiomeric dopants was successfully expressed at the macroscopic level *via* the principle of hierarchical self-assembly.

The mechanism underlying the helical curling of macrostripes was explained by a multi-scale chemo-mechanical theoretical model (Fig. 10c). Morphology characterization revealed that the microscopic structures of the outside surface and inside surface of the stripes were different because the outside surface was closed to the solution while the insider surface was attached to the polypropylene substrate. The morphologies of fibrous assemblies are uniformly single-handed (due to the chiral dopants) for the outside surface, but almost straight (due to the restriction of the substrate) for the inside surface. Considering that iPrOH is a poorer solvent than THF, the introduction of iPrOH triggered the intermolecular shrinkage of the stripes. Due to the different morphologies of the fibrous assemblies between the inside and outside surfaces, microscopic shrinkage strains on these two surfaces were also different. Specifically, the normal and shear strain simultaneously pulled the flat strip, resulting in both bending and torsional effects. These two different strains followed different orientations of the fibrous assemblies on the inside and outside surfaces, thus inducing the helical chirality of the stripes at the macroscale level. The feature size of the helicity of the macrostripes depended on the intensity of shrinkage strain, while the latter parameter was controlled by the concentration of iPrOH. Therefore, both the handedness and the intensity of helical macrostripes could be controlled easily by the hierarchical self-assembly process.^139^

In addition, the helical self-motion of the macrostripes was observed upon the stimuli of another enantiomeric molecule. For example, the colour of the stripe immediately changed from green to blue accompanied by left-handed helical motion when exposed to (*S*)-(+)-2-aminohexane, while the colour change was slow after exposure to (*R*)-(−)-2-aminohexane and the deformation of the stripe was random.^139^ This enantioselective discrimination behaviour of the macrostripe indicated that the hierarchical order was conducive to enhancing the responsive sensitivity and accuracy of chiral functional materials.

### 4.7 Biomimetic synthesize chiral structures

**Fig. 11:**
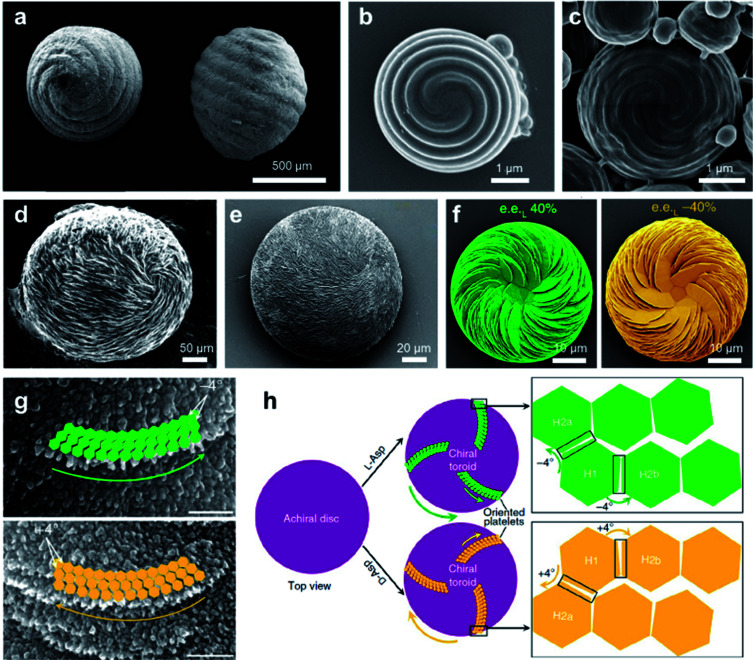
Biomimetic synthesize hierarchical chiral structures *via* self-assembly. (a) Charophyte gyrogonites from the Huchpayacu formation, Contamana, and Peruvian Amazonia in (left) basal and (right) lateral views. Reproduced from ref. 144 with permission from Elsevier Ltd, copyright 2016. (b and c) SEM images of particles with opposite chiral spiral morphologies. (b and c) Reproduced from ref. 145 with permission from American Chemical Society, copyright 2018. (d) Rounded, chiral pathologic calcium carbonate vaterite otoconia from the inner ear of an aged. Reproduced from ref. 147 with permission from Elsevier Ltd, copyright 1982. (e) Synthetic chiral suprastructure of hierarchically organized vaterite induced by the amino acid l-Asp as prepared in the lab. Reproduced from ref. 146 with permission from American Association for the Advancement of Science, copyright 2018. (f) SEM images of vaterite helicoids showing chiral vaterite platelets having counterclockwise or clockwise orientation when l- or d-Asp is the majority enantiomer under nonracemic solution conditions, respectively. Reproduced from ref. 149 with permission from Nature Publishing Group, copyright 2019. (g) High-magnification SEM images of chiral toroid platelets showing tilting of nanoparticle (nanohexagon) growth induced by selective chiral amino-acid adsorption. This chirality-inducing effect causes rounded/curved platelet edge growth having either a counterclockwise direction for l-Asp (green arrows) or a clockwise direction for d-Asp (yellow arrows). (h) Tilting mechanism leading to the formation of chiral vaterite toroids. (g and h) Reproduced from ref. 148 with permission from Nature Publishing Group, copyright 2017.

Chiral structures are ubiquitous in nature, ranging across multiscale and imparting various functions.^140,141^ As a result of millions of years of evolution, hierarchical helical structures in biological systems are usually fabricated by combining several size scales, which is difficult to synthesize or replicate.^142,143^ Nevertheless, the biomimetic synthesis of hierarchical chiral structures has attracted substantial attention due to their structural functions and their potential applications (see Section 5 for a more detailed discussion). Fig. 11a shows the pictures of the seeds and their protective inorganic shells of charophyte (a type of green alga).^144^ This spiral morphology was recently achieved artificially by a simple direct emulsification process of polypeptides.^145^ In detail, the polypeptides contained a majority of γ-benzyl glutamate (bE) residues, which showed an α-helical conformation. Depending on the polypeptide, the spiral morphology on the surface of most particles had a raised part and a depressed part with a cross-sectional width ranging from 200 to 600 nm. As anticipated, the direction of spiral structures was controlled by the chirality of polypeptides, and particles produced from the emulsification of l residue-rich polypeptides exhibited right-handed spirals (clockwise), while those from d residue-rich polypeptides had left-handed spirals (counterclockwise). However, the formation of spirals could be suppressed when polypeptides with an opposite α-helical conformation were added, thus indicating the important role of a well-defined α-helical secondary structure in self-assembly. Another interesting point is that the best-defined spirals were produced from copolypeptides instead of the model α-helix homopolypeptides. The mechanism for the formation of microscopic helices (spirals) on the surface of particles is not yet fully understood, one hypothesis being due to the folded fibril bundles of coiled helical polypeptides during a hierarchical self-assembly process.^145^

Another typical example of biomimetic synthesize is the 3D homochiral suprastructures of biomineral calcium carbonate (Fig. 11e),^146^ whose morphology remarkably resembles pathologic chiral vaterite otoconia found in the human inner ear (Fig. 11d).^147^ The formation of such chiral hierarchically organized structures was induced simply by chiral amino acid through a nanoparticle tilting growth mechanism.^148^ In the absence of amino acids, calcium carbonate only formed rhombohedral calcite crystals, which were achiral. Depending on the form of the amino-acid additive, hierarchically toroid-shaped chiral superstructures that resembled the blades of a propeller or a pinwheel were formed. Notably, only acidic amino acids such as aspartic (Asp) and glutamic (Glu) acids induced chiral superstructures, while neutral and basic chiral amino acids such as alanine and lysine only induced symmetrically hexagonal, nontoroidal and achiral structures.

The chiral direction of these toroid-shaped suprastructures was controlled by the chirality of the amino acid additives. A counterclockwise (right-handed) spiralling morphology was induced by l-enantiomers, whereas d-enantiomers induced a clockwise (left-handed) morphology. The racemic mixture of amino acids only resulted in symmetric vaterite with no chiral character. More recent work showed that a mixed nonracemic amino acid system also induced the same chiral spiralling morphology, indicating a chirality dominance effect (Fig. 11f).^149^ High-resolution microscopy demonstrated that both achiral (no additive) and spirally oriented curved platelets (with amino acid) had a nanoparticle substructure (Fig. 11g). Due to the low concentrations of calcium (2.5 mM) and carbonate ions (0.5 mM), no pre-existing nanoparticles were observed in the reaction solutions. Based on the experimental and simulation results, the underlying mechanism is illustrated in Fig. 10h. In the absence of amino acids, straight hexagonal platelet edges were formed due to the perfect orientation of nanoparticles. In contrast, the presence of chiral amino acid at the surface of the nanoparticles broke the perfectly oriented nucleation/attachment, and a slight misaligned tilt (about 4°) was formed between two adjacent nanohexagonal prisms (Fig. 11g and h). The direction of this imperfect oriented nucleation was controlled by the purity of adding chiral amino acids. With the further growth and extension of vaterite platelets, the small misaligned difference was amplified, resulting in a chiral hierarchical superstructure of calcium carbonate.^148^

### 4.8 Chiral structures of emerging materials

In the above sections, we mainly discuss chiral nanostructures that contain either organic or inorganic components. The development of self-assembled hybrid materials, such as chiral metal–organic frameworks (MOFs), recently has attracted significant attention due to their unique properties and devious potential applications.^150–152^ MOF materials are a subclass of coordination polymers and are generally constructed by coordination-driven self-assembly of organic ligands and metal ions or clusters. One of the special features of MOFs is that they are often well-defined porous structures with precise positions and orientations of interacting moieties. The present nanocavities in chiral MOFs thus provide space-restricted chiral microenvironments, which are extremely sensitive to asymmetric guests. Therefore, chiral MOFs and their derivatives usually show good performance in chiral separation, enantioselective recognition and supramolecular asymmetric catalysis.^7,150,153–158^

The construction of chiral metal–organic assemblies also follows the basic principle that has been discussed in Section 3. They can be formed by using either chiral organic ligands as building blocks or chiral induction from chiral templates. Only a few cases show that completely achiral building blocks can form chiral MOFs.^159,160^

**Fig. 12:**
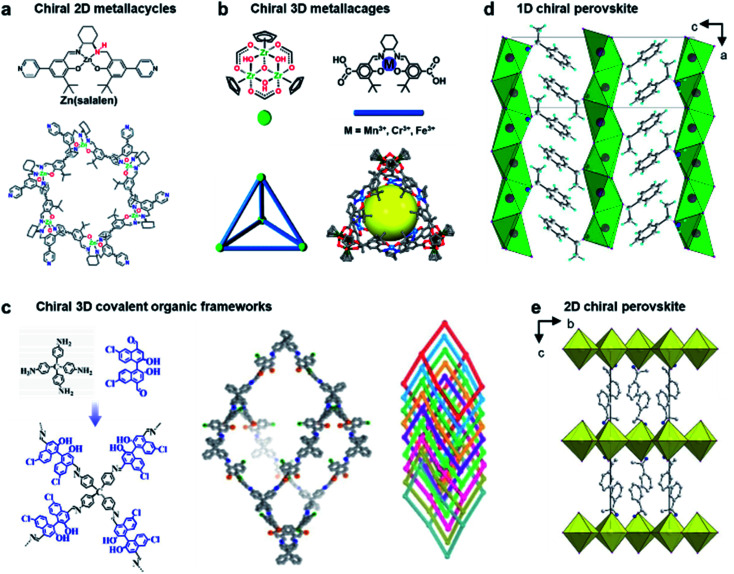
Chiral structures of emerging materials. (a) Self-assembly of chiral NH-controlled supramolecular 2D metallacycles from Zn(salalen). Reproduced from ref. 163 with permission from American Chemical Society, copyright 2017. (b) Self-assembly and crystal structure of chiral metallosalen-based 3D cages. Reproduced from ref. 165 with permission from American Chemical Society, copyright 2018. (c) Molecular structures and structural representations of chiral 3D covalent organic frameworks. Reproduced from ref. 174 with permission from Wiley-VCH, copyright 2021. (d) Packing diagram of ((*S*)-C_6_H_5_C_2_H_4_NH_3_) [PbBr_3_], viewed along the b axis. Reproduced from ref. 181 with permission from Wiley-VCH, copyright 2003. (e) Packing diagram of the chiral hybrid PbI_4_((*R*)-C_6_H_5_CH(CH_3_)NH_3_)_2_. There are two complete inorganic layers in the unit cell. Reproduced from ref. 182 with permission from American Chemical Society, copyright 2006.

Due to crystalline networks, MOFs usually have intrinsic limitations on solubility. In contrast, metal–organic complexes (MOCs) also have well-defined structures but they are discrete and thus can be dissolved in solution (soluble porous units of MOFs); this feature has appreciable contribution to expand the potential application of metal–organic assemblies.^7,161,162^Fig. 12a shows an example of 2D metallacycles controlled by chiral NH functionalities.^163^ Considering the importance of hydrogen bonding in molecular recognition and biological processes, chiral hydrogen bonding sites were introduced in the design of chiral ligands. As shown from the molecular coordination structures, the 2D metallacycles containing six free chiral NH groups emitted cyan fluorescence under UV-light.

Interestingly, as fluorescent chiral receptors, these 2D metallacycles enabled enantioselective discrimination of α-hydroxycarboxylic acids, amino acids and amines in solution through fluorescence quenching. Moreover, chiral drugs such as l-dopa and d-penicillamine were also detected through fluorescence enhancement in a crystalline state. This enantioselective fluorescence was explained by a donor–acceptor electron-transfer mechanism.^163^ This work highlighted the important role of chiral NH functionalities, which significantly improved the selectivity and binding affinity in the chiral recognition and discrimination of biomolecules.

In addition to 2D metallacycles, 3D metal–organic cages have more complex structures due to increased binding/active sites. To date, chiral metal–organic assemblies mainly contained just one type of ligand and metal ion, while most of the metalloenzymes in living systems, such as catalase and nitrogenase iron protein, have been constructed from multinuclear metal complexes.^7,164^ The heterometallic active sites thus enable a biochemical process through enzymes with high efficiency. Inspired from natural systems, Fig. 12b shows that a single coordination cage can contain two different catalytically active sites by a rational molecular design and hierarchical self-assembly strategy.^165^ 3D metal–organic chiral cages are prepared by using six dicarboxylate ligands derived from enantiopure Mn(salen), Cr(salen) and/or Fe(salen) as linear linkers and four *C*_3_-symmetric Cp_3_Zr_3_ clusters as three-connected vertices. The resulting nanocage is a tetrahedral-shaped structure, the internal tetrahedral volume of the cage being 795 Å^3^.

Due to the high energy of the Zr–O bond (776 kJ mol^−1^), the trinuclear zirconium clusters remarkably improved the structural stability in acidic, neutral, and weak basic aqueous solutions. In addition, the manipulation of catalytic metal species in the nanocage was precisely controlled through the mixed-linker strategy. The high stability and heterometallic active sites enabled the 3D nanocages to be an efficient supramolecular catalyst for sequential asymmetric reactions with enantiomeric excess up to 99.9%.^165^ This work demonstrated that the activity and selectivity of metal–organic assemblies could be tuned by organic linkers, and the mixed-linker with multiple active sites provided a new platform to engineer novel supramolecular assemblies and materials for enantioselective processes.

Chiral covalent organic frameworks (COFs) are a new class of chiral porous materials.^8,166–168^ Instead of the coordination interaction between metals and organic linkers, COFs are generally constructed from only organic molecules through strong covalent bonds and reticular chemistry.^169,170^ Due to the stable covalent interaction, COF materials exhibit several outstanding features such as highly ordered pore architectures, high electrical conductivity, structural diversity, *etc.*^171–173^ Similar to metal–organic assemblies, COF materials can be rationally designed with specific structures and functions by selecting different building blocks.^11^ For example, 3D COFs can be utilized as the template to enhance the enantioselectivities of molecular catalysts (Fig. 12c).^174^ These chiral COFs were prepared through imine condensation of tetrahedral tetraamine and chiral molecular catalysts (1,1′-binaphthol dialdehydes). Note that the molecular catalysts in a homogeneous state showed no enantioselectivity for the asymmetric acetalization of aromatic aldehydes and 2-aminobenzamide. However, once they were incorporated into the conformationally rigid pores of 3D COFs, the enantioselectivity was significantly boosted with up to 93% yield and 97% ee. The transformation from being completely nonselective to highly selective was attributed to the steric hindrance and confinement effect of the 3D framework. In addition, the COF catalysts exhibited high stability and could be recycled multiple times without clear decay.^174^ By enantioselective induction under a confined microenvironment, this work provided a new approach to designing and constructing asymmetric catalysts from non-enantioselective catalysts.

Perovskite, which is used to describe the structure of calcium titanium oxide, now represents an important class of hybrid organic–inorganic materials featuring AMX_3_ formula, where A represents a monovalent cation (*e.g.*, NH_2_CHNH_2_^+^, CH_3_NH_3_^+^, and Cs^+^), M is a metallic cation (*e.g.*, Pb^2+^ and Sn^2+^), and X represents a halogen (*e.g.*, Cl^−^, Br^−^, and I^−^).^175^ Metal halide perovskites usually have flexible crystal structures and tuneable composition due to hybrid organic–inorganic interaction, thus paving a way for various potential applications, particularly in photovoltaics and optoelectronics.^176–180^

The first example of a chiral perovskite was a 1D chiral-perovskite single crystal that was reported in 2003 (Fig. 12d),^181^ and later 2D chiral perovskite crystals were synthesised in 2006 (Fig. 12e).^182^ After this pioneering research, various chiral perovskite materials have been developed with diverse properties.^183^ The chiral nanostructures of perovskites range from 1D to 3D.^184^ Meanwhile low-dimensional perovskites generally contain a higher percentage of chiral ligands, thereby displaying a higher degree of chirality. In addition, compared to 3D perovskites, the 2D analogues are more resistant to humidity during the fabrication of devices and measurement.^185,186^ Moreover, lower-dimensional perovskites are easy to process, showing a remarkable structural tunability and flexibility (see more details in Section 5.2).^14,187^

## 5. Application of chiral nanoassemblies

Although chiral molecules themselves exhibit their intrinsic functions, nanoassemblies achieved through hierarchical self-assembly often show advanced functions which are inaccessible for single chiral building blocks. In addition, the dynamic nature of chiral nanoassemblies can give rise to numerous novel properties that differ from those of common chiral materials. In this section, recent successful applications of chiral nanostructures are summarized.

### 5.1 Bio-related functions

In terms of nanotechnology, nanoparticles (NPs) are one of the most prominent and promising candidates for bio-related applications. Hierarchical chiral self-assembly provides a scalable and versatile way to regulate individual NPs into sophisticated chiral nanostructures.^2,188–190^ On the other hand, the construction of nanoparticle assemblies is usually directed by biocompatible chiral components such as amino acids, peptides, proteins and DNA.^2,191–193^ In particular, the DNA origami methodology is widely used to build complex and unusual structures with high fidelity because of the sequence-specific base-pairing and exceptional programmability.^192,193^

**Fig. 13:**
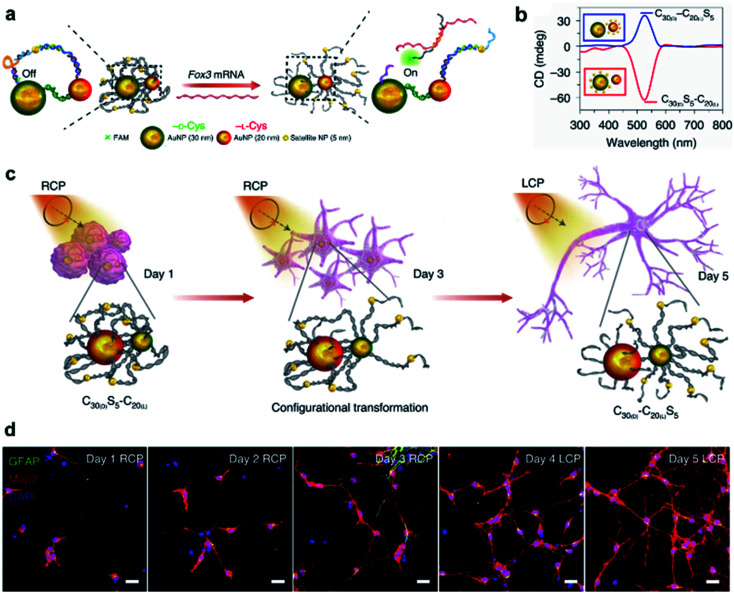
CPL-accelerated neural stem cell differentiation *via* chiral nanoassemblies. (a) Schematic of Fox3 mRNA triggering the configuration transformation from C_30(d)_S_5_–C_20(l)_ to C_30(d)_–C_20(l)_S_5_. (b) CD spectra of these two nanoassemblies in phosphate buffered saline (PBS). (c) Schematic of differentiation of NSCs with CPL after daily incubation with C_30(d)_S_5_–C_20(l)_ for five days. (d) Confocal images of NSCs incubated with C30_(d)_S5–C20_(l)_ for five days. Red, Map2 for mature neurons; blue, DAPI for nuclei; green, GFAP for astrocytes. Scale bars, 20 μm. Reproduced from ref. 196 with permission from Nature Publishing Group, copyright 2021.

Due to the high polarizability, chiral inorganic nanoparticle assemblies generally have strong interactions with circularly polarized light (CPL).^2,194,195^ By using this edge, a recent example showed that circularly polarized photons transduced by chiral nanoassemblies were endowed with ability to accelerate the differentiation of neural stem cells into neurons (Fig. 13).^196^ Three types of Au NPs with different sizes (30 nm, 20 nm, and 5 nm) were firstly modified with enantiopure cysteine and further formed chiral assemblies by using single-stranded DNA. The 5 nm NPs (satellite NPs) could closely attach to either 30 nm or 20 nm NPs, namely C_30(X)_S_5_–C_20(X)_ and C_30(X)_–C_20(X)_S_5_, respectively, where X represents the handedness of cysteine coated on the surface of NPs. Note that the DNA linker between 5 nm and 30 nm NPs was complementary to Fox3 mRNA, the latter being expressed during neural stem cell (NSC) differentiation into neurons. Therefore, such a reconfiguration process enabled entanglement between chiral nanoassemblies and nanofilaments of the cytoskeleton inside the cell (Fig. 13a).

Moreover, the reconfiguration process also induced an inversion of the CD signal (Fig. 13b). For example, C_30(d)_S_5_–C_20(l)_ showed a negative peak in the CD spectrum, but turned positive after hybridizing with Fox3 mRNA. Note that the chiroptical activity arose from the mirror asymmetry of the entire superstructure instead of individual nanoparticles. Experimental results indicated that the differentiation rate of neural stem cells with C_30(d)_S_5_–C_20(l)_ remarkably increased when irradiated with right-handed circularly polarized light compared to when irradiated with left-handed or linearly polarized light, and *vice versa*. Since the chiroptical polarity of chiral nanoassemblies changed during reconfiguration, the cell differentiation was further improved by switching the polarity of light during the differentiation process (Fig. 13c and d). The concept of circularly polarized light-accelerated differentiation was further utilized in the therapy of Alzheimer's disease.^196^ It should be noted that the DNA-bridged constructs and the tight binding way could also be accomplished by other means and adjusted accordingly. Nevertheless, this work emphasized the important role of chiral nanoassemblies in the field of biological applications, and the synergistic effect of chirality, flexibility and biocompatibility was highlighted.

### 5.2 Chiral optics and electronics

**Fig. 14:**
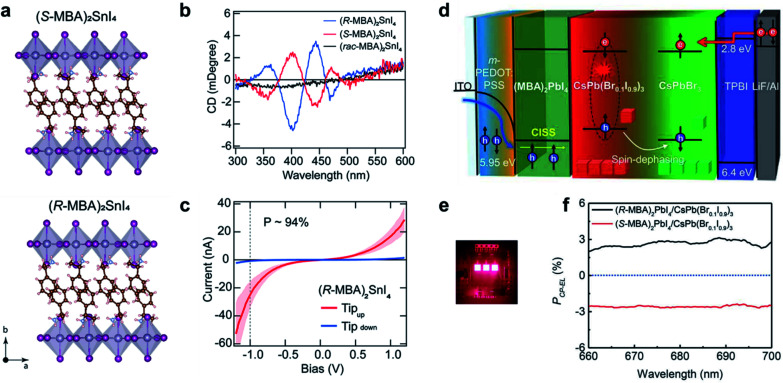
Chiral-induced spin selectivity of metal-halide perovskite. (a) Crystal structure and (b) CD spectra of (*R*-MBA)_2_SnI_4_ and (*S*-MBA)_2_SnI_4_. (c) Room temperature *J*–*V* curves of (*R*-MBA)_2_SnI_4_, which shows a spin polarization of up to 94%. (a–c) Reproduced from ref. 203 with permission from American Chemical Society, copyright 2020. (d) Schematic illustration of CP-EL from mixed halide perovskite nanocrystals. (e) Operating device image of spin-LEDs. (f) PCP-EL of spin-LEDs based on a CISS layer/CsPbBr_3_ NC heterostructure. (d–f) Reproduced from ref. 14 with permission from American Association for the Advancement of Science, copyright 2021.

Hybrid organic–inorganic halide perovskites have recently attracted attention due to their revolutionary tuneable properties, in particular photovoltaic and emergent optoelectronic applications. Adding new degrees of complexity, for instance chirality to these organic/inorganic hybrid semiconductors, opens the door to various exciting properties and functionalities.^197–200^ Since the chirality of organic components can be effectively transferred to the inorganic sublattice, the resulting chiral hybrid materials are promising candidates for nonlinear optics, spin-polarized LEDs and ferroelectricity.^184,201–204^Fig. 14a–c show new 2D tin iodide layered perovskites using chiral and achiral methylbenzylammonium (MBA) cations as ligands.^203^ The crystal structure contains a layer of corner-sharing SnI_6_^4−^ octahedra and a bilayer of organic MBA cations (Fig. 14a). Note that the organic chiral layer separates the inorganic layers, resulting in a chiral space group *P*2_1_2_1_2_1_ for (*R*-/*S*-MBA)_2_SnI_4_. Transmission circular dichroism spectra show distinct CD signals from 300 to 500 nm, which can be attributed to the inorganic Sn–I sublattice (Fig. 14b). The spin polarized properties were further investigated by magnetic conductive-probe atomic force microscopy (mCP-AFM). Due to the chiral-induced spin selectivity (CISS) effect, the vertical charge transport through the oriented chiral thin films was highly spin-dependent, with a spin-polarization as high as 94%.

The superior spin polarized charge transport of chiral perovskites was recently employed to create optoelectronic devices. Typical optoelectronic technologies and devices establish on the regulation of the charge of current carriers instead of their spin, while the creation and investigation of spin-polarized light sources, such as spin-polarized lasers and spin-polarized light-emitting diodes (spin-LEDs), is one important topic in the development of spintronic devices. For spin-LED devices, the key step is the injection of pure spin-polarized charge carriers into the active region.^205,206^ Traditionally, spin polarization is realized with the help of magnetic fields or polarized ferromagnetic contacts. Recently, chiral-induced spin selectivity, an effect that electron transmission through chiral molecules is spin dependent,^197,207^ was used to produce spin-polarized carriers instead of magnetic fields or ferromagnetic contacts (Fig. 14d–f).^14^ A spin polarized hole injection layer was prepared from a chiral 2D layered metal-halide perovskite referred to as (*R*-/*S*-MBA)_2_PbI_4_. The spin orientation was determined by the chiral organic molecules in this layer. The recombination of the injected spin-polarized holes and electrons occurred in the colloidal perovskite nanocrystal emitting layer, resulting in circularly polarized electroluminescence (CP-EL). Due to the limitation of spin scattering in the nanocrystals, the CP-EL polarization degree (*P*_CP-EL_) was around ±0.25% in the range of 660 to 740 nm. By introducing a mixed-halide NC-emitting layer (Fig. 14d), the optimized spin-LED displayed ±2.6% circularly polarized electroluminescence at room temperature (Fig. 14f).

### 5.3 Asymmetric synthesis

The rational design of asymmetric catalysts is the key topic in asymmetric reactions. Although great success has been achieved in chiral molecular catalysts, efficient supramolecular assemblies capable of asymmetric catalysis have emerged in recent years.^6,208–213^ One particular example is chiral porous materials (MOFs and COFs), as mentioned in Section 4.8. Here, we stress that the efficiency of asymmetric supramolecular catalysis is closely related to the morphology of nanostructures.

**Fig. 15:**
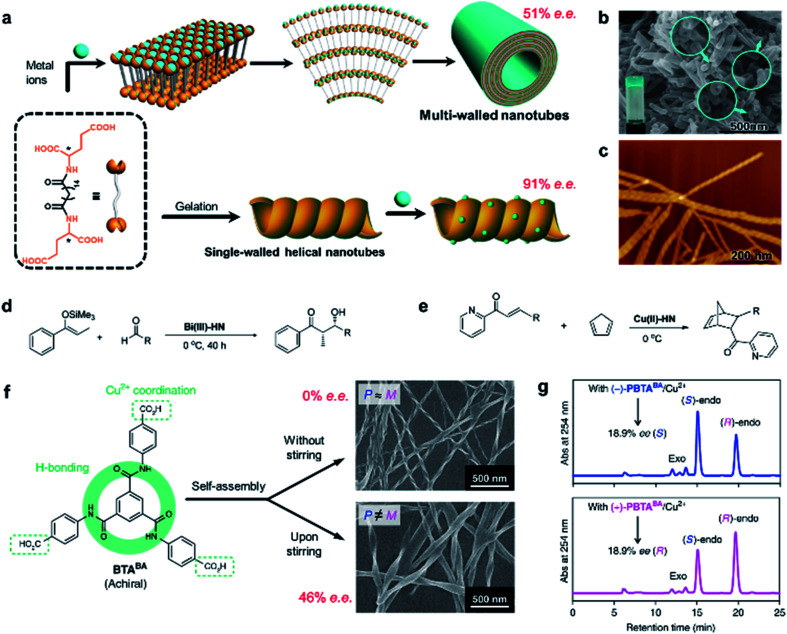
Self-assembled chiral nanostructures as scaffolds for asymmetric reactions. (a) Schematic illustration of the hierarchical self-assembly of multi-walled nanotubes (top) and single-walled helical nanotubes (bottom). SEM and AFM image of the (b) multi-walled nanotubes and (c) single-walled helical nanotubes, respectively. (b) Reproduced from ref. 214 with permission from American Chemical Society, copyright 2011; (c) reproduced from ref. 215 with permission from American Chemical Society, copyright 2016. Asymmetric (d) Mukaiyama aldol reaction and (e) Diels–Alder reaction. (f) Stirring induced symmetry-broken helical nanoribbons. (g) Chiral HPLC traces after the nanoribbon catalysed Diels–Alder reaction. (f and g) Reproduced from ref. 216 with permission from Nature Publishing Group, copyright 2019.

As shown in Fig. 15a, an l-glutamic acid-based gelator was co-assembled with copper(ii) ions to form multi-walled nanotubes.^214^ The addition of Cu^2+^ ions could not only stabilize the nanostructures but also be utilize as the centre for catalytic reactions. Specifically, the Cu^2+^ ions connected two neighbouring molecules and firstly formed a multilayer. Because of the chiral packing of the lamella structures, the layer structure rolled into nanotubes, while the chirality was transferred from the l-glutamic acid into the whole nanotube including the Cu^2+^ ions. The Cu^2+^-coordinated nanotubes gave an enantiomeric excess of 52% in the Diels–Alder reaction. This is the first example of using self-assembled metal–organic nanotubes to catalyze asymmetric reactions.

However, the subsequent study found that the same gelator alone could self-assemble into a helical single-wall nanotube in water (Fig. 15c), and this helical morphology was preserved when adding an appropriate amount of metal salt.^215^ Interestingly, the single-walled helical nanotubes exhibited a remarkably enhanced enantioselectivity and reactivity, and achieved 91% enantiomeric excess in the Diels–Alder reaction within 60 min. More importantly, such helical single-wall nanotubes could coordinate with different metal ions to catalyze the corresponding reactions. For example, if Cu^2+^ was replaced with Bi^3+^, the self-assemblies could catalyze Mukaiyama aldol reactions with enantiomeric excess up to 97%.^215^ Therefore, this work provided a general strategy to construct supramolecular asymmetric catalysts.

It should be mentioned that the enantioselectivity disappeared once the chiral nanoassemblies were destroyed (*e.g.*, adding excess NaOH). Although the build blocks were chiral themselves, the chiral centre at the molecular level did not show any enantioselectivity. In addition, the asymmetric reactions were also accelerated by supramolecular nanoassemblies. These results confirmed that the stereochemical selectivity of helical nanotubes was important for the efficiency of asymmetric catalysis.

Besides chiral building blocks, certain achiral components are known to form nanostructures with a chiral geometry (see the discussion in Section 3). A recent example showed that symmetry-broken helical assemblies composed of completely achiral organic molecules could serve as a chiral ligand to catalyse asymmetric reactions (Fig. 15f).^216^ Helical nanoribbons were prepared by applying magnetic rotary stirring during the self-assembly of a benzene-1,3,5-tricarboxamide derivative. Similarly, the helical nanoribbons coordinated with Cu^2+^ ions and were then employed for the Diels–Alder reaction. After the optimization of the reaction condition, the enantiomeric excess eventually reaches 46% with 99% conversion (Fig. 15g).

### 5.4 Display

**Fig. 16:**
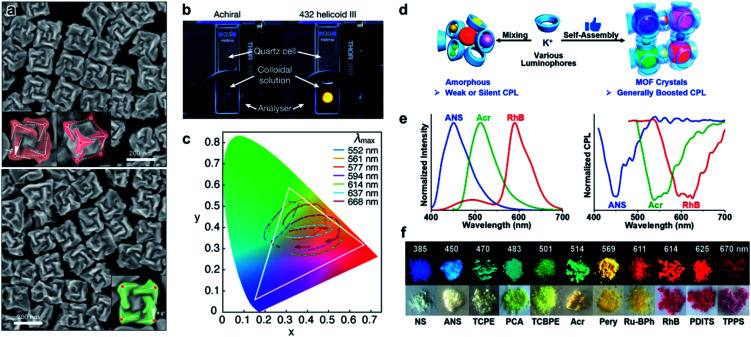
Chirality induced colour modulation. (a) SEM images of three-dimensional plasmonic helicoids controlled by l-Cys (up) and d-Cys (down). (b) Photographs of achiral and 432 helicoid III solutions, showing the light transmitted under cross-polarized conditions. (c) Colour transition patterns of 432 helicoid III nanoparticles traced on CIE (CIE, International Commission on Illumination). Each pattern shows elliptical traces with a clockwise rotational direction that reflects the asymmetric colour transition. (a–c) Reproduced from ref. 217 with permission from Nature Publishing Group, copyright 2018. (d) Illustration of the boosted CPL from lum@γCD-MOF. (e) FL and CPL spectra of various luminophores. (f) Photographs of various CPL crystals of lum@γCD-MOF under the irradiation of 365 nm (upper) and visible light (lower). (d–f) Reproduced from ref. 152 with permission from Wiley-VCH, copyright 2020.

A large number of structural colour responses have been observed in biological organisms with selective reflection of left- or right-handed circularly polarized (CP) light. Understanding the role of chirality in light manipulation thus becomes important and necessary. Fig. 16a presents the SEM images of gold nanoparticles with a three-dimensional chiral structure.^217^ The optical activity, handedness and chiral plasmonic resonance of these nanoparticles were controlled by amino acids and peptides. As a result of the highly twisted chiral structures (as shown in SEM images), the chiral nanoparticles showed a strong circular dichroism signal, and the dissymmetry factor (*g*-factor) was as high as 0.2 at 622 nm, which was the largest value among any other chiral nanostructures fabricated through a bottom-up strategy. Due to the large *g*-factor, the macroscopic colour of the helicoid nanoparticles could be easily manipulated by changing the polarization. For example, a bright yellow colour of chiral nanoparticle solution was observed when using a cross-polarized polarizer, while the achiral nanoparticles didn't show any transmission (Fig. 16b). Moreover, different from the symmetric patterns for achiral nanoparticles, the colour transition of chiral nanoparticles was continuous and asymmetric when rotating the analyser (Fig. 16c), which provided a versatile method of chirality-dependent colour modulation.

In contrast to the transmitted colour modulation as shown in Fig. 16a–c, circularly polarized luminescence (CPL) defined by the differential emission of left- and right-circularly polarized light from chiral systems reflects the chiral properties of the excited state. Generally, a large number of molecules with both chiral and luminescent units are needed to achieve full-colour CPL. However, the CPL activity of the obtained chiral emitters is unpredictable and a tedious and long synthesis procedure is inevitable. Fortunately, the introduction of the self-assembly strategy provides a new way for the fabrication of CPL materials.^15^ For example, as shown in Fig. 16d, numerous achiral luminophores within a range of 0.5 to 2.1 nm could be integrated into the chiral space of γ-cyclodextrins (γCD) and exhibited significantly boosted CPL compared with the amorphous powders of γCD and luminophores.^152^ The chirality came from the cubic chirality of the metal–organic framework (γCD-MOF) that self-assembled from γ-CD and alkali metal ions. It should be noted that various luminophores, regardless of the charge (neutrally, positively or negatively) and aggregated luminescence state (ACQ or AIE), can be efficiently integrated into γCD-MOF, resulting in full-colour CPL materials with tuneable capability.

## 6. Conclusions and outlook

The manipulation of chiral structures by programming complex molecular interactions and external environments is one of the most challenging topics in supramolecular chemistry. As discussed in this review, with regard to both the construction methods and potential applications of chiral nanostructures, considerable progresses have been made. Through hierarchical self-assembly, chiral structures at multiscale levels can be fabricated, accompanying by abundant applications in various fields.

On the molecular scale, chirality mostly arises from a tiny configuration difference, but can lead to the helical organization of assemblies at microscopic and/or macroscopic levels. Understanding the transmission and amplification of chirality through space and across length scales is of growing importance not only in supramolecular chemistry and materials science but also in biology and physics. Therefore, more effort should be devoted to uncovering the underlying mechanism.

Chiral nanostructures are not necessarily made up of chiral units, and the co-assembly of chiral and achiral components has been found to play a crucial role in the field of chiroptic and optoelectronic materials,^14,15,218–220^ in which their properties could be tuned by different chirality. For example, incorporating chiral organic ligands into metal-halide perovskites leads to chiral hybrid semiconductors with fascinating spin-dependent properties (Fig. 14).^14^ In addition, chiral supramolecular structures are considered as excellent matrices and templates to regulate the assembly of inorganic building blocks,^221^ endowing hybrid assemblies with compelling chiroptic performances, such as chiral plasmonic and circularly polarized emission.^2,15,190,192,222–224^ Therefore, utilizing the hierarchical self-assembly strategy to combine chiral molecules and other functional materials is a promising research direction as the chirality might endow the hybrid assemblies with interesting and exciting properties.

Recent progress in supramolecular chemistry indicates that the self-assembly process is governed by kinetics, and out-of- (or far-from-) equilibrium states are becoming topical.^99,100,102,121^ Such processes are usually accompanied by the morphological change of chiral nanostructures, thus giving rise to vast functional potential. In particular, far-from-equilibrium assemblies, which need a continuous supply of energy to persist (like some biological supramolecular machinery), are a step forward in imitating the multi-scale, multifunctional and hierarchically organized structures in nature.

## Author contributions

Y. S. and M. L. proposed, designed, prepared and revised the review together.

## Conflicts of interest

There are no conflicts to declare.

## Supplementary Material
